# High Frequency of *Thermodesulfovibrio* spp. and *Anaerolineaceae* in Association with *Methanoculleus* spp. in a Long-Term Incubation of *n*-Alkanes-Degrading Methanogenic Enrichment Culture

**DOI:** 10.3389/fmicb.2016.01431

**Published:** 2016-09-16

**Authors:** Bo Liang, Li-Ying Wang, Zhichao Zhou, Serge M. Mbadinga, Lei Zhou, Jin-Feng Liu, Shi-Zhong Yang, Ji-Dong Gu, Bo-Zhong Mu

**Affiliations:** ^1^State Key Laboratory of Bioreactor Engineering and Institute of Applied Chemistry, East China University of Science and TechnologyShanghai, China; ^2^School of Biological Sciences, The University of Hong KongHong Kong, China; ^3^Shanghai Collaborative Innovation Center for Biomanufacturing TechnologyShanghai, China

**Keywords:** alkanes degradation, long-chain alkanes, microbial community, 16S rRNA gene, *Thermodesulfovibrio*, *Anaerolineaceae*, *Methanoculleus*, methanogenesis

## Abstract

In the present study, the microbial community and functional gene composition of a long-term active alkane-degrading methanogenic culture was established after two successive enrichment culture transfers and incubated for a total period of 1750 days. Molecular analysis was conducted after the second transfer (incubated for 750 days) for both the active alkanes-degrading methanogenic enrichment cultures (T2-AE) and the background control (T2-BC). A net increase of methane as the end product was detected in the headspace of the enrichment cultures amended with long-chain *n*-alkanes and intermediate metabolites, including octadecanoate, hexadecanoate, isocaprylate, butyrate, isobutyrate, propionate, acetate, and formate were measured in the liquid cultures. The composition of microbial community shifted through the successive transfers over time of incubation. Sequences of bacterial and archaeal 16S rRNA gene (16S rDNA) and *mcrA* functional gene indicated that bacterial sequences affiliated to *Thermodesulfovibrio* spp. and *Anaerolineaceae* and archaeal sequences falling within the genus *Methanoculleus* were the most frequently encountered and thus represented the dominant members performing the anaerobic degradation of long-chain *n*-alkanes and methanogenesis. In addition, the presence of *assA* functional genes encoding the alkylsuccinate synthase α subunit indicated that fumarate addition mechanism could be considered as a possible initial activation step of *n*-alkanes in the present study. The succession pattern of microbial communities indicates that *Thermodesulfovibrio* spp. could be a generalist participating in the metabolism of intermediates, while *Anaerolineaceae* plays a key role in the initial activation of long-chain *n*-alkane biodegradation.

## Introduction

Quantitatively, alkanes are one of the most significant component of petroleum hydrocarbons (Head et al., 2010). They were long considered to be recalcitrant to biodegradation in the absence of molecular oxygen, nitrate, and/or sulfate (Heider et al., 1998). In recent years, alkanes biodegradation via methanogenesis has been a topic of increasing interests thanks to the first report of a successful enrichment culture converting long-chain alkanes (specifically hexadecane) to methane (Zengler et al., 1999). Many relevant researches on methanogenic alkanes-degradation studies appeared afterward (Anderson and Lovley, 2000; Jones et al., 2008; Gray et al., 2011; Wang et al., 2011; Li et al., 2012; Mbadinga et al., 2012; Zhou et al., 2012; Aitken et al., 2013; Cheng et al., 2013b; Berdugo-Clavijo and Gieg, 2014; Embree et al., 2014; Sherry et al., 2014; Liang et al., 2015). The initial activation of alkane degradation under anaerobic conditions may involve fumarate addition to the parent alkane, hydroxylation/carboxylation and, in some specific cases, intra-hydroxylation (Callaghan, 2013). Among the above mentioned activation mechanisms, addition to fumarate appear as the most prevalent and almost the best characterized mechanism in anaerobic hydrocarbon degradation (Aitken et al., 2013). The gene *assA*/*masD* coding the alkylsuccinate synthetase has eventually been considered as a valuable biomarker for detecting fumarate addition pathway in alkane degradation (Callaghan et al., 2010).

Microbial communities capable of degrading petroleum hydrocarbons under methanogenic conditions are often complex consortia, at least consisting of various fermenting bacteria, syntrophic bacteria and methanogens at least. Generally, the constitution of microbes from different hydrocarbons impacted environments like aquifers, sediments, and soils can be dramatically different. However, among them some microbial taxa appeared with relatively high frequency, a good example of this is that many researches shared the similar microorganisms members of the *Syntrophaceae* (*Smithella*/*Syntrophus*) which was inferred to have the ability of degradation of alkane (Bakermans and Madsen, 2002; Kasai et al., 2005; Allen et al., 2007; Gray et al., 2011; Ramos-Padron et al., 2011; Cheng et al., 2013a; Embree et al., 2014; Tan et al., 2014a,b). A survey that collated published 16S rRNA gene data from 26 culture-independent analyses of methanogenic hydrocarbon impacted environments showed that bacterial sequences affiliated with *Firmicutes* were detected at the highest frequency followed by *γ*-*proteobacteria, δ*-*proteobacteria, 𝜀*-*proteobacteria, β*-*proteobacteria, Bacteroidetes, Actinobacter, α*-*proteobacteria, Chloroflexi, Thermotogae, Nitrospira, Spirochaetes, Acidobacter, Planctomycetes* and OP11 (Gray et al., 2010). So far, at least 19 anaerobic, alkane-oxidizing microorganisms have been isolated (Webner, 2012; Khelifi et al., 2014; Schouw et al., 2016), while 17 of the isolated strains were affiliated with the phylum of *Proteobacteria*, other two strains belongs to *Firmicutes* and *Archaeoglobales*, respectively. The large proportion of non-cultivable and metabolically inactive organisms interferes with the identification of the active responsible for the degradation. In addition, the various syntrophic associations in methanogenic consortia and the obligate anaerobic conditions make isolation of them in pure cultures difficult and impossible with the current available techniques. Consortium obtained through enrichment culturing with long-term stability for methanogenic alkane degradation can eliminate the inactive members considerably so that the essential ones can be accumulated. In the present research work, a methanogenic alkanes-degrading consortium was established after enrichment culturing and long-term of incubation amended with a mixture of *n*-alkanes (C_15_–C_20_) as the sole sources of carbon and energy. The succession pattern of microbial communities together with the diversity and abundance of potential functional genes were analyzed via PCR based clone libraries construction coupled with quantitative real-time PCR analysis in this study. The degradation intermediates and the final product methane were measured during degradation at the same time.

## Materials and Methods

### Enrichment Cultures

Inoculum was obtained from an initial methanogenic enrichment culture from Menggulin petroleum reservoir production water (Huabei oilfield, China) amended with *n*-alkanes as described previously (Li et al., 2012). About 10 ml (20%) of inoculum were transferred into an autoclaved serum bottle (internal volume 120 ml) containing 50 ml of sterilized basal medium prepared by the Hungate technique (Bryant, 1972) and then sealed with a butyl rubber stopper (Bellco Glass, Inc., Vineland, NJ, USA) and aluminum crimp seal. The basal medium composition was described elsewhere (Wang et al., 2012). Active enrichment cultures were amended with the mixture of *n*-alkanes (C_15_–C_20_) as the sole carbon and energy sources (three replicates). Autoclaved controls were prepared in the same way but sterilized three times (three replicates). Background controls were prepared without addition of any organic carbon source (three replicates). The mixture of *n*-alkanes (C_15_–C_20_) contained *n*-pentadecane (C15; ≥99%), *n*-hexadecane (C16; ≥99%), *n*-heptadecane (C17; ≥99%), *n*-octadecane (C18; ≥99%), *n*-nonadecane (C19; ≥99%), and *n*-eicosane (C20; ≥99%) (Sigma–Aldrich, Milwaukee, WI, USA). About 30 μl of the *n*-alkanes mixture were added to each of the empty serum bottles except the background controls under a stream of N_2_ gas before sterilization. All of the cultures were incubated at 37°C in the dark.

### Chemical Analysis

Gas chromatography (GC) was used to measure the production of methane in the headspace gas of serum bottle during the incubation. Two hundred microliters of the headspace gas taken by gas-tight syringe were injected onto GC by a micro-syringe for analysis. Program setting of the GC analysis was: the initial column temperature at 60°C for 12 min, then increased to 200°C at a rate of 15°C/min, the final temperature at 200°C sustained for 24 min. Temperature of injector and flame ionization detector (FID) was maintained at 200°C. An external standard curve of methane was used for converting peak areas of methane into their respective concentrations (*R*^2^ = 0.994, *n* = 6).

Gas Chromatography-Mass Spectrometer (GC-MS; Agilent Technologies, Inc.) was used for the detection of residual *n*-alkanes and intermediate metabolites in the aliquot phase through the incubation. For the analysis of intermediate metabolites containing mainly long-chain fatty acids (LCFAs) and volatile fatty acids (VFAs), 5 ml of culture aliquot were taken and the pH was adjusted with ammonia water to >12 and then dried in an oven at 110°C. Esterification was performed by adding 0.5 ml of 10% butanol/sulfate solution at 90°C for 60 min. Extraction of LCFAs and VFAs was conducted with 0.5 ml of *n*-hexane and 0.5 ml *n*-dodecane, respectively, and the extracts were injected onto the GC-MS. For LCFAs analysis, program setting was oven temperature maintained at 120°C for 3 min, increased at the rate of 8°C/min to 260°C for 10 min while for VFAs, oven temperature was maintained at 60°C for 1 min and then increased at the rate of 15°C/min to 130°C. Residual *n*-alkanes in the aliquot samples were extracted with *n*-hexane as described previously (Wang et al., 2011).

### DNA Extraction and PCR Amplification

At the end of incubation period, 10 ml of active culture (three replicates) and the background control (three replicates) were withdrawn from each serum bottle, then centrifuged at 1200 × *g* for 10 min. The biomass pellet after centrifugation was used for DNA extraction by using AxyPrep^TM^ Bacterial Genomic DNA Maxiprep Kit (Axygen Biosciences, USA) according to the manufacturer’ instructions.

The universal primer sets of 8F/805R (Savage et al., 2010) and 340F/1000R (Gantner et al., 2011) were used for bacterial and archaeal 16S rRNA gene amplification, respectively. For bacterial 16S rRNA gene, PCR amplification reaction was performed according to the followings: 5 min for initial denaturation at 95°C, followed by 38 cycles of 95°C for 30 s, 52°C for 45 s, 72°C for 60 s, and a final elongation step at 72 C for 10 min. For archaeal 16S rRNA gene, PCR amplification conditions were as follows: 5 min for initial denaturation at 95°C, followed by 10 cycles of 95°C for 30 s, 60°C for 30 s (decreased by 0.5°C per cycle to 50°C), 72°C for 60 s. After touchdown, 30 additional cycles at annealing temperature of 50°C were performed, followed by the final elongation step at 72°C for 10 min.

Alkylsuccinate synthetase genes (*assA*) and methyl coenzyme-M reductase genes (*mcrA*) as the functional genes in the process of methanogenic degradation of alkanes were also investigated. PCR primer sets of assA2F/assA2R (Callaghan et al., 2010) and ME3MF/ME2R (Hales et al., 1996; Nunoura et al., 2008) were used for the PCR amplification, respectively. PCR thermal cycles for *assA* were carried out as follows: 5 min for initial denaturation at 95°C, followed by 10 cycles of 95°C for 30 s, 60°C for 30 s (decreased by 0.5°C per cycle to 55°C), 72°C for 60 s. After touchdown, 28 additional cycles at annealing temperature of 55°C were performed, followed by the final elongation step at 72°C for 10 min. PCR cycles for *mcrA* were as follows: 5 min for initial denaturation at 95°C, followed by 38 cycles of 95°C for 30 s, 52°C for 45 s, 72°C for 60 s, and a final elongation step at 72°C for 10 min.

### Construction of 16S rRNA Gene, and *assA* and *mcrA* Functional Gene Libraries

After the PCR products were gel purified by using Gel Extraction Kit (Axygen Biosciences, USA), the purified DNA fragments were cloned into *E. coli* using pMD19^®^-T simple vector kit (TaKaRa Bio Inc., Japan). The white clones were picked randomly into 1 ml of Luria Broth (LB) medium amended with ampicillin and incubated for 24 h at 37°C. PCR primer set M13-47 (5′-CGCCAGGGTTTTCCCAGTCACGAC-3′) and RV-M (5′-GAGCGGATAACAATTTCACACAGG-3′) was used for positive clone detection. Sequencing of the positive clones was accomplished on an ABI 377 automated sequencer. Chimeric sequences of 16S rRNA gene sequences were excluded by Bellerophon (Huber et al., 2004). Valid sequences with more than 97% similarity were classified through the BLASTclust of MPI bioinformatics toolkit (Biegert et al., 2006) to arrive operational taxonomic unit (OTU). Functional genes were translated through ExPASY translation tool^1^. Sequences were compared to the GenBank Nucleotide Sequence Database using BLAST (Altschul et al., 1990) to identify the nearest matches in the GenBank database. Phylogenetic and molecular evolutionary analyses were conducted using MEGA6.0 software (Tamura et al., 2013) with neighbor-joining method (Saitou and Nei, 1987) and 1000 bootstrap replicates.

### Quantitative PCR

Quantitative PCR of target genes were performed by using SYBR Green I Real-Time PCR (BioRad CFX96 thermocycler, Bio-Rad Laboratories Inc., USA). We amplified 16S rRNA gene of *Archaea* and *Bacteria*, each target gene was amplified with specific primers ARC787F/ARC1059R (Yu et al., 2005) and BAC338F/BAC805R (Yu et al., 2005), respectively. These two genes were amplified separately in a SYBR Green I Real-Time PCR reaction with a 10-fold dilution series of plasmids containing target DNA sequences as a calibration standard for establishing the standard curve. Quantitative PCR reaction (20 μl) was composed of SYBR Green Realtime PCR Master Mix-Plus (10 μl; TaKaRa Bio Inc., Japan), Plus Solution 2 μl (TaKaRa Bio Inc., Japan), PCR primers (1 μl of each), 4 μl of sterile water, and 2 μl DNA template (27.4 ng/μl for T2-AE and 15.8 ng/μl for T2-BC). The conditions were as follows: pre-denaturation for 3 min at 95°C, followed by 38 cycles of denaturation at 94°C for 20 s, annealing temperature was 60°C and 57°C, respectively, for 30 s, elongation at 72°C for 60 s.

### Nucleotide Sequences Accession Numbers

The sequences generated in this study were deposited in GenBank under accession numbers KP109826-KP109908 and KP341767-KP34176769.

## Results

### Headspace Methane and Intermediate Metabolites

**FIGURE 1:**
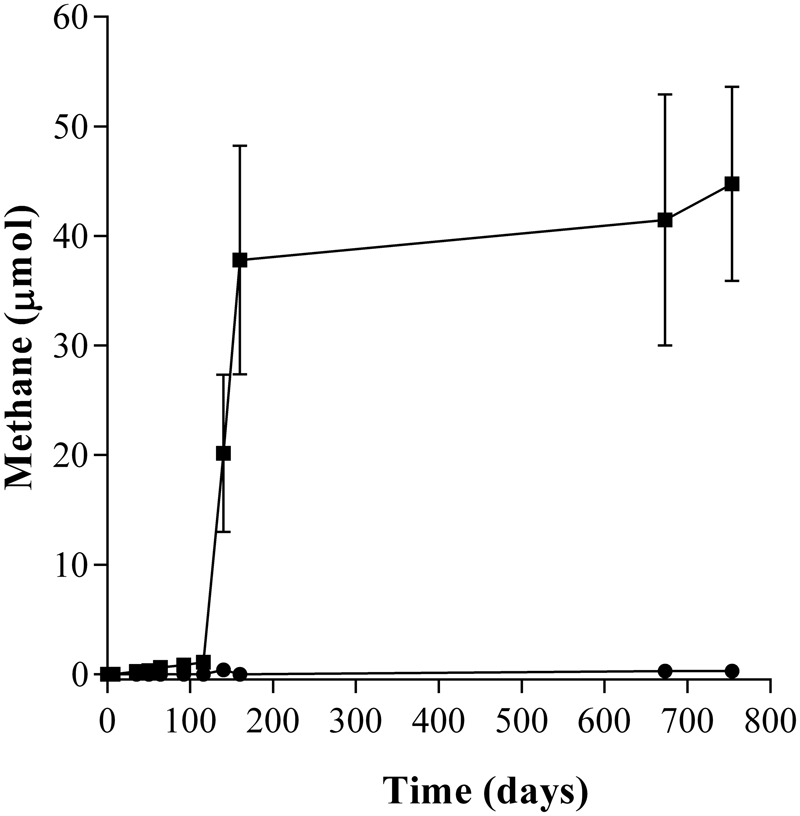
**Methane production of the *n*-alkanes degradation consortium under methanogenic conditions.** (■) incubated with *n*-alkanes mixture (C_15_–C_20_) as the sole sources of carbon and energy (T2-AE; three replications), and (●) without any *n*-alkanes and other carbon sources as the background control cultures (T2-BC; three replicates).

During the incubation of the second enrichment transfer, the lag phase in the amended enrichment cultures (T2-AE) was only 92 days and 44.76 μmol of methane were generated while in the background control without amendment of any alkanes (T2-BC) approximately 0.28 μmol of methane were detected (**Figure 1**). Intermediate metabolites including LCFAs (Supplementary Figure S1) and VFAs (Supplementary Figure S2), octadecanoate, hexadecanoate, isocaprylate, butyrate, isobutyrate, propionate, acetate, and formate were detected in the enrichment cultures only. Quantity of residual *n*-alkanes were detected through the GC-MS with cetyl chloride as the surrogate standard. The net consumption of total *n*-alkanes was 53.31 μmol by subtracting the residual *n*-alkanes in the active enrichment cultures from autoclaved controls. Stoichiometry of methane production showed methane accumulated in headspace accounted for 6.23% of theoretically predicted value (Supplementary Table S1).

### Phylogenetic Analysis of Bacteria

**FIGURE 2:**
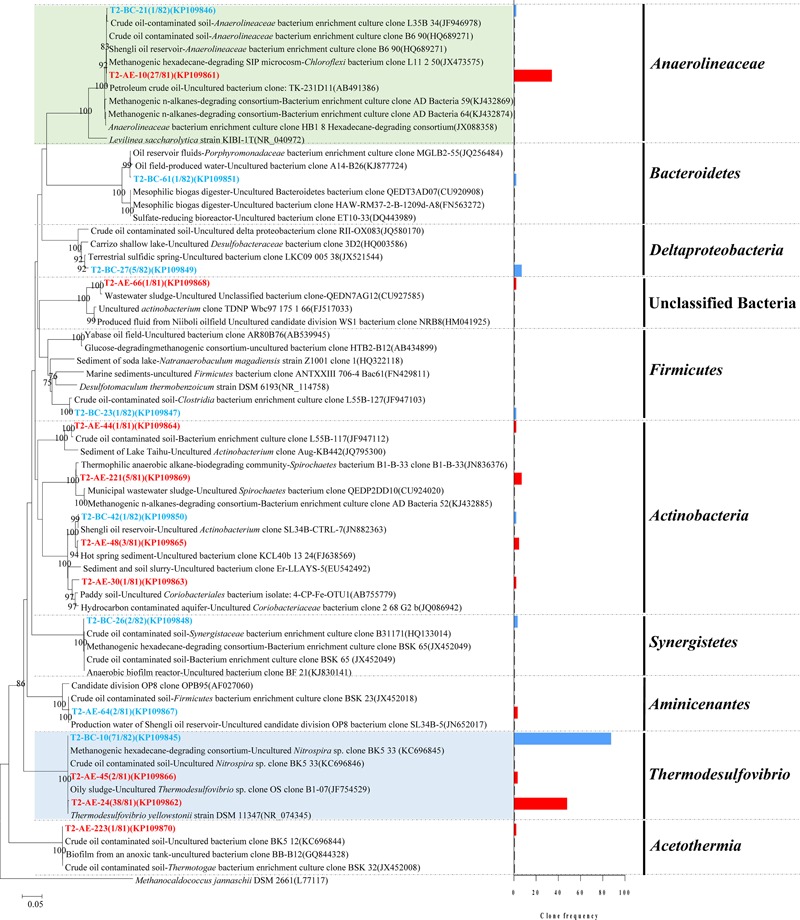
**Phylogenetic tree of bacterial 16S rRNA gene sequences from methanogenic alkanes-degrading enrichment cultures (T2-AE; in red) and background control cultures (T2-BC; in blue).** The tree is rooted with *Methanocaldococcus jannaschii* DSM 2661 (L77117) as the out group. The OTUs are shown with clone names and accession numbers. One-thousand bootstraps were performed with neighbor-joining method. Bootstrap values below 75% are not shown. Frequency of clones is shown in the bar graph.

A total of 100 positive clones were picked for sequencing bacteria 16S rRNA genes from both the active enrichment (T2-AE) and background control cultures (T2-BC). After removing vector parts and checking chimeras, 81 and 82 valid sequences, we obtained for the active culture and the control, respectively. The valid sequences were classified through BLASTclust of MPI bioinformatics toolkit (Biegert et al., 2006) with 97% similarity and 10 OTUs for T2-AE and seven OTUs for T2-BC were obtained (**Figure 2**). The OTU of “T2-AE-24 (KP109862)” which contains the most clone sequences belongs to *Thermodesulfovibrio* within the phylum of *Nitrospirae* in the active enrichment cultures (T2-AE). Followed by the OTU of “T2-AE-10 (KP109861)” belongs to *Anaerolineaceae* within the phylum of *Chloroflexi* which covers 27 clone sequences. Bacteria in the genus of *Thermodesulfovibrio* having the highest abundance constituted 49% of all of the 81 clones (**Figure 2**). Family *Anaerolineaceae* as the dominant bacteria made up 33%. *Acetothermia, Aminicenantes, Actinobacteria*, and unclassified bacteria composed the remaining 18% (**Figure 2**). In T2-BC, OTU of “T2-BC-10 (KP109845)” affiliated with *Thermodesulfovibrio* within the phylum of *Nitrospirae* representing the most clone sequences of 71 in the whole 82 valid clones (**Figure 2**). Bacteria in the genus of *Thermodesulfovibrio* were the most dominant bacteria of 87%, while *Anaerolineaceae* occupied 1%. The remaining 12% comprised *Actinobacteria, Bacteroidetes, Actinobacteria, Synergistia*, and *Firmicutes* (**Figure 2**).

### Phylogenetic Analysis of Archaea

**FIGURE 3:**
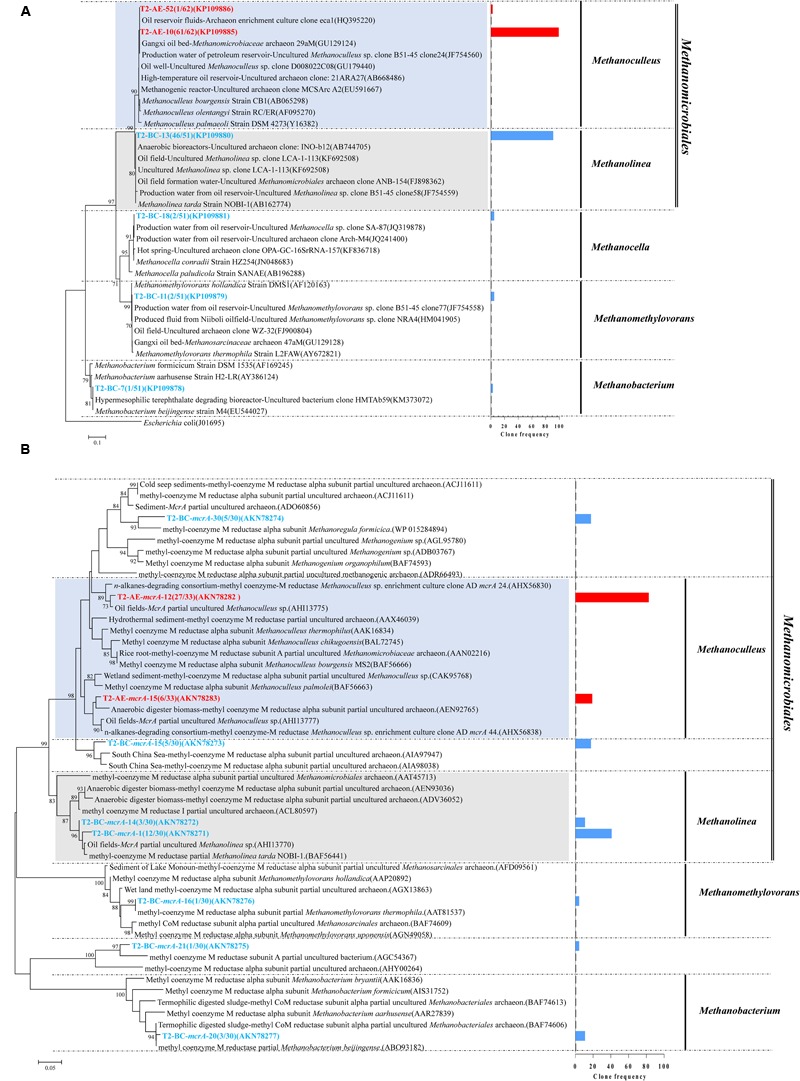
**Phylogenetic tree of archaeal 16S rRNA gene sequences from methanogenic alkanes-degrading enrichment cultures (T2-AE; in red) and background control cultures (T2-BC; in blue).** This tree is rooted with outgroup sequence from *Escherichia coli* (J01695) as an outgroup. The OTUs are shown with clone names and accession numbers. Topology of the tree was obtained by the neighbor-joining method. One-thousand time bootstraps testing were performed. Bootstrap values below 70% are not shown. Frequency of clones are shown in the bar graph **(A)**; Phylogenetic tree of deduced amino acid sequences of methyl coenzyme-M reductase genes (*mcrA*) from methanogenic alkanes-degrading enrichment culture (in red) and background control cultures (T2-BC; in blue). Topology of the tree was obtained by the neighbor-joining method. The evolutionary distances were computed using the Poisson correction method. One-thousand time bootstraps testing were performed. Bootstrap values below 70% are not shown **(B)**.

Positive clones were also picked for sequencing archaea 16S rRNA gene. A total of 61 and 51 valid sequences were obtained for the active enrichment (T2-AE) and background control cultures (T2-BC), respectively, after the removal of vector and chimeras checking. Valid sequences were analysis through BLASTclust of MPI bioinformatics toolkit (Biegert et al., 2006) with 97% similarity to generate the OTUs. There were two OTUs for active enrichment cultures (T2-AE) and four OTUs for background control cultures (T2-BC) (**Figure 3A**). For the active enrichment cultures (T2-AE), the OTU of “T2-AE-10 (KP109885)” contains the majority 61 clones of the total 62 clones. All of the 62 clone sequences belong to *Methanoculleus* within the *Methanomicrobiales* order (**Figure 3A**). For the background control (T2-BC), the OTU of “T2-BC-13 (KP109880)” had 46 of the total 51 clones. This OTU affiliated with *Methanolinea* in the order of *Methanomicrobiales* occupying 90% of all valid clone sequences. *Methanobacterium, Methanocella*, and *Methanomethylovorans* constituted the remaining 10% of the clone library (**Figure 3A**).

### Diversity and Phylogenetic Analysis of *assA* and *mcrA* Genes

**FIGURE 4:**
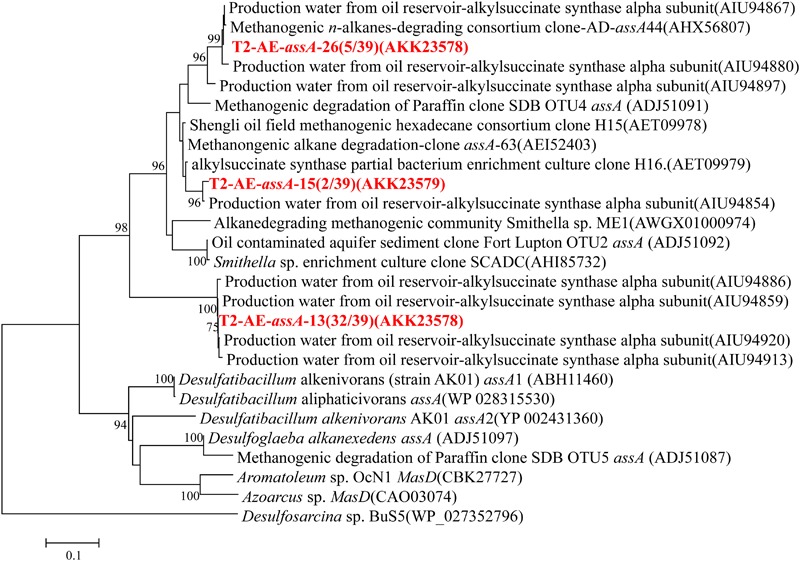
**Phylogenetic tree of deduced amino acid sequences of alkylsuccinate synthetase genes (*assA*) genes from methanogenic alkanes-degrading enrichment culture (in red).** The topology of the tree was obtained by the maximum likelihood method. Bootstrap values (*n* = 1000 replicates), values below 75% are not shown.

Functional gene of *assA* was only detected in the active enrichment cultures (T2-AE) by using the primer set of assA2F/assA2R (Callaghan et al., 2010). Valid sequences were used for phylogenetic analysis and resulted in three OTUs at 97% similarity (**Figure 4**). Most sequences were in the OTU of “T2-AE-*assA*-13 (AKK23578)” which contains 32 of the total 39 clone sequences. This OTU has a 100% similarity with the sequence (AIU94859) from production water of an oil reservoir of Jiangsu oil field (Bian et al., 2015). Background control cultures (T2-BC) generated no detectable products by PCR reactions.

Both the T2-AE and T2-BC cultures detected the functional gene *mcrA* by using the prime set of ME3MF/ME2r (Nunoura et al., 2008) and obtained two and seven OTUs, respectively. All of the clone sequences in T2-AE cultures affiliated with *Methanoculleus* within the order of *Methanomicrobiales* (**Figure 3B**). This result was coincident with archaeal 16S rRNA gene clone library. The OTU of “T2-AE-*mcrA*-12 (AKN78282)” showed 97% similarity with the sequence from the *n*-alkanes-degrading consortium (AHX56830). Except the OTU of “T2-BC-*mcrA*-21 (AKN78275),” *mcrA* functional gene sequences in T2-BC cultures had three clear classifications: *Methanomicrobiales, Methanobacteriales*, and *Methanosarcinales* (**Figure 3B**).

### Quantitative PCR

16S rRNA genes of *Archaea* and *Bacteria* were amplified for both T2-AE and T2-BC to detect the whole archaea and bacteria 16S rRNA gene copies. Gene abundance (gene copies per milliliter of culture aliquot) of *Archaea* was 4.17 × 10^7^ (copies/ml) and 1.01 × 10^6^ (copies/ml) in T2-AE and T2-BC, respectively. Gene abundance (gene copies per milliliter of culture aliquot) of *Bacteria* was 1.48 × 10^7^ (copies/ml) and 1.62 × 10^6^ (copies/ml) for T2-AE and T2-BC, respectively. For *Archaea* and *Bacteria*, 16S rRNA gene qPCR reaction, amplification efficiency was 105.6% and 102.3%, *R*^2^-values was greater than 0.989 and 0.986, respectively.

## Discussion

The entire duration of nearly 5 years of enrichment culturing included the first transfer (about 950 days) and a subsequent second transfer (over 750 days), both of the two transfers and incubations received the mixture of *n*-alkanes (C_15_–C_20_) as the sole carbon and energy sources. During the incubation, the variations of microbial community in different enrichment transfers were investigated. Microbial community of the Menggulin petroleum reservoir production water sample (OS) and the initial transfer of incubation after 400 days (T1-400) were analyzed by our laboratory and reported previously (Li et al., 2012). In the current research, microbial communities in the second transfer from both the active enrichment cultures (T2-AE) and the background control (T2-BC) were investigated after 750 days of incubation.

### High Frequency of *Thermodesulfovibrio* and *Anaerolineaceae*

**FIGURE 5:**
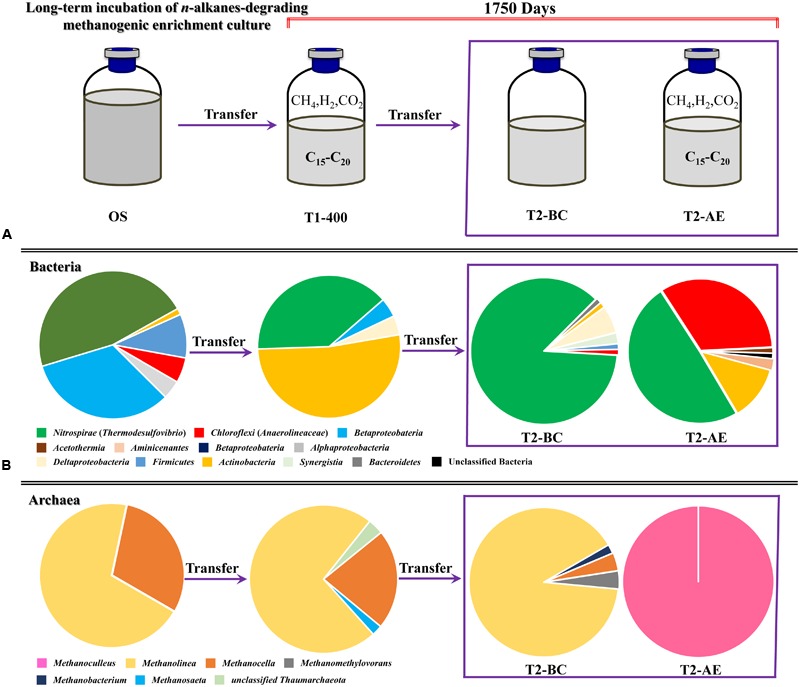
**The microbial community varied with the time of incubation from the analysis of bacterial and archaeal 16S rRNA gene clone libraries.** Relative proportion of bacterial lineages **(A)** and archaeal lineages **(B)** during the methanogenic alkanes-degrading enrichment culture. “OS” represents the initial inoculum came from Huabei oilfield petroleum reservoir production water sample in China; “T1-400” represents the initial enrichment culture at 400 days which added with *n*-alkanes (C_15_–C_20_) for details see Li et al. (2012) (initial transferred enrichment culture lasted for 950 days); “T2-AE” and “T2-BC” represent the active enrichment cultures and background control cultures, respectively, in the second transferred enrichment culture amended with *n*-alkanes as the sole sources of carbon and energy for 750 days.

Clear variation of bacteria community was observed after the 5 years of methanogenic alkanes degrading enrichment culturing (**Figure 5**). In the original petroleum reservoir production water sample (OS), the dominant bacteria were *Gammaproteobacteria* (46.6%) and *Betaproteobacteria* (32.9%). After nearly 400 days of incubation with addition of a mixture of *n*-alkanes (T1-400) initially, *Actinobacteria* (52.2%) and *Thermodesulfovibrio* (39.1%) were detected as the dominant bacteria. It is clear that the period of *Actinobacteria* as the dominant bacteria only maintained for a short period of time at the beginning. Then the abundance of *Actinobacteria* decreased sharply to 6.2% as a minority after another 1350 days of incubation (including a second transfer of incubation for 750 days) in T2-AE, whereas *Thermodesulfovibrio* remained the most frequently encountered genus and increased gradually to 49.4%. At the same time, *Anaerolineaceae* rose conspicuously to 33.3% as the dominant bacteria. Similar results were also observed in our earlier *n*-alkanes-degrading consortium originated from an oily sludge (Liang et al., 2015). Thus, *Thermodesulfovibrio* and *Anaerolineaceae* as the most dominant bacteria implies a major role they play in the long-term methanogenic alkanes-degrading consortium.

*Thermodesulfovibrio* was the only overwhelming majority (86.6%) in the control cultures (T2-BC). *Anaerolineaceae* occupied only 1.2%, a significant difference from the 33.3% in T2-AE sample. Since both T2-AE and T2-BC were the second enrichment transfers of incubation from the same inoculum initially, such major difference in consortium composition should be attributed to the amendment of organic carbons. When the microorganisms were subject to a starvation condition (T2-BC), abundance of bacteria like *Anaerolineaceae* and *Actinobacteria* dropped because of the lack of organic carbons available. Comparing to the background control T2-BC, the dominant *Anaerolineaceae* in the active enrichment transfer culture T2-AE should be closely related to the alkanes-degrading process. The total bacteria in T2-AE experienced an obvious surge comparing to T2-BC according qPCR analysis showing the alkanes-degrading activity.

*Thermodesulfovibrio* within the phylum *Nitrospirae*, is a sulfate-reducing microorganism (SRM) that could use sulfate as the terminal electron acceptor in their energy metabolism (Sherry et al., 2013). OTU of *Thermodesulfovibrio* in this research shared the highest identity (100%) with *Thermodesulfovibrio yellowstonii* strain DSM 11347^T^ (NR_074345), which is able to use lactate, pyruvate, and hydrogen as electron donors in the presence of sulfate (Henry et al., 1994). Interestingly, the medium had no sulfate available, and methane was the main end product. Theoretically *Thermodesulfovibrio*-like species could cooperate with other bacteria or methanogens syntrophically in the alkanes-degrading processes. The presence of this bacterium in similar environments is also reported by others (Sekiguchi et al., 1998; Magot et al., 2000; Pérez-Jiménez et al., 2001; Roest et al., 2005; Li et al., 2007, 2012; Mbadinga et al., 2012; Cheng et al., 2013b; Liu et al., 2014).

Since the general appearance as the majority both in T1-400, T2-AE, and T2-BC, *Thermodesulfovibrio* as a generalist may participate in the intermediate metabolism, such as the degradation of fatty acids. While *Anaerolineaceae* as a specialist should be a key player in the degradation process and may likely take part in the initial activation of alkane for degradation since the obvious increase in T2-AE in clear contrasting with OS, T1-400 and T2-BC. This seems logic because many researches have implied the potential of alkane degradation. *Chloroflexi* was firstly reported to be involved with toluene degradation in a 10-year toluene-degrading methanogenic consortium. Several researches also showed evidence that *Anaerolineaceae* was one of the frequently encountered microbial species in petroleum hydrocarbon conditions (Winderl et al., 2008; Savage et al., 2010; Gray et al., 2011; Sherry et al., 2013; Sutton et al., 2013; Liang et al., 2015). In an enrichment culture metabolizing low-molecular-weight alkanes (*n*-propane and *n*-pentane) under mesophilic sulfate-reducing conditions, *Anaerolineaceae* as one of the community members showed the potential of hydrocarbon degradation for this lineage organisms (Savage et al., 2010). In a recent study of methanogenic biodegradation of short-chain *n*-alkanes revealed that members of the *Anaerolineaceae* may either be directly involved in activation and biodegradation of *n*-octane and *n*-decane or act as scavengers of metabolic intermediates (Shahimin et al., 2016). Thus, comparative analysis of 16S rRNA genes sequenced from the cultures with and without alkanes amendment indicated that members of the family *Anaerolineaceae*, which constitutes more than 33% of the bacterial community 16S rRNA genes in *n*-alkanes amended culture, may play a key role in the anaerobic metabolism of long-chain alkanes in the long-term incubation.

The obligate anaerobic, non-photosynthetic and multicellular filamentous family *Anaerolineaceae* in *Chloroflexi* phylum contains seven isolated strains in the five genera (Sekiguchi et al., 2003; Yamada et al., 2006, 2007; Grégoire et al., 2011). When comparing *Anaerolineaceae* in T2-AE (KP109861) with the isolated strain, the highest identity is only at 91% with *Levilinea saccharolytica* strain KIBI-1^T^ (NR_040972), indicating that this *Anaerolineaceae* in petroleum hydrocarbon environment may be a new division different from the isolated strains. Interestingly, *Anaerolineaceae* clone (KP109861) in this research has a very high identity (99%) with the *Anaerolineaceae* clone (KJ432869) in our former *n*-alkanes-degrading consortium from oily sludge, suggesting that they should be in the same division within the family of *Anaerolineaceae*.

### *Methanoculleus* in the Methanogenic Process from Long-Term Incubation

Methanogens in the long-term methanogenic alkanes-degrading enrichment culture experienced sharp changes especially in T2-AE sample (**Figure 5**). Both archaeal 16S rRNA gene clone library and functional gene *mcrA* clone library showed that the only methanogen in T2-BC sample was *Methanoculleus*. In contrast, methanogens in T2-BC were mostly *Methanolinea* (90.2%) and others were *Methanobacterium, Methanocella*, and *Methanomethylovorans* which was consistent with the archaeal community in the sample of OS and T1-400 while *Methanoculleus* was undetected. The phylum *Euryarchaeota* contains six methanogenic orders: *Methanomicrobiales, Methanobacteriales, Methanococcales, Methanosarcinales, Methanopyrales*, and *Methanocellales* (Garrity and Holt, 2001; Ferry, 2010). Both genera of *Methanolinea* and *Methanoculleus* are in *Methanomicrobiales* order. The succession of *Methanolinea* and *Methanoculleus* suggests a competitive growth relationship between these two hydrogenotrophic methanogens. Comparing to the isolated methanogens, the OTU of *Methanoculleus* in T2-AE shows a 99% identity with *Methanoculleus receptaculi*, while, OTU of *Methanolinea* in T2-BC has a 99% identity with *Methanolinea tarda*. *M. receptaculi* was reported to take acetate as the growth factor with minimal doubling time of 8.2 h (Cheng et al., 2008). *M. tarda* can use acetate, yeast extract or Coenzyme M stimulates as growth factors with minimal doubling time of 144 h (Imachi et al., 2008). Since acetate and a faster growth rate than *Methanolinea* was detected in T2-AE, it is natural that *Methanoculleus* finally turned to be the most dominant methanogens in T2-AE sample.

### Possible Metabolic Pathway of Methanogenic Degradation of *n*-Alkanes

**FIGURE 6:**
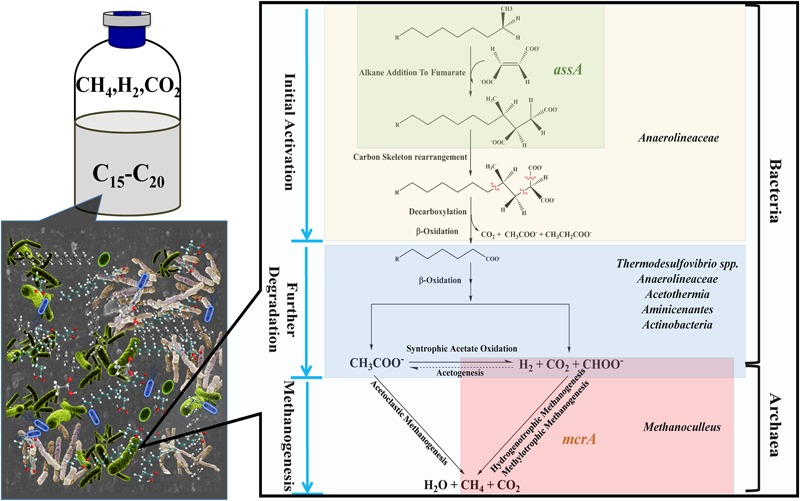
**Possible metabolic pathway of methanogenic degradation of *n*-alkanes, based on the pathway proposed by Wilkes et al. (2002) and Callaghan et al. (2012).** In the methanogenic *n*-alkanes-degrading culture, metabolic pathway from *n*-alkanes to methane can be presumed. Fumarate addition could be involved in the initial activation of alkanes since the detection of *assA* functional gene. In the phase of methanogenesis, hydrogen, carbon dioxide, and formate were produced through syntrophic acetate oxidation pathway, followed by the production of methane through hydrogenotrophic or methylotrophic methanogenesis. Microorganisms are positioned according their probable role within microbial consortia.

The presence of *assA* functional gene in the active enrichment cultures (T2-AE) suggests that fumarate addition should be involved in the initial activation of alkanes in this methanogenic alkanes-degrading culture (**Figure 6**). Moreover, the non-detectable *assA* functional gene in the background control (T2-BC) confirmed the activity of *assA* functional gene in T2-AE sample. By contrast, the total amount of *Bacteria* and *Archaea* gene copies in T2-AE sample were much higher than those in T2-BC by quantitative PCR analysis. All of these results demonstrate the methanogenic alkanes-degrading activities in T2-AE sample. After initial activation, alkanes converted to LCFAs and VFAs, including octadecanoate, hexadecanoate, isocaprylate, butyrate, isobutyrate, propionate, acetate, and formate. And this intermediate metabolites-degrading phase should be responsible mostly by *Chloroflexi* (more specifically, *Anaerolineaceae*) since the community genomic analyses indicated that most members in the phylum of *Chloroflexi* are anaerobic acetogenic microbes which also have the genomes of complete Wood-Ljungdahl pathway and beta-oxidation of saturated fatty acids (Hug et al., 2013). Growth of some *Methanoculleus* members requires acetate even though they do not convert it to methane (Barret et al., 2015) and they were reported to produce methane from H_2_/CO_2_ and from formate (Oren, 2014). Thus, in methanogenic phase, *Methanoculleus* as the methanogens generated methane through hydrogenotrophic methanogenesis or methylotrophic methanogenesis (**Figure 6**).

## Conclusion

The obvious increase of *Thermodesulfovibrio* and *Anaeroline-aceae* in alkanes-dependent methanogenic culture after a long-term of incubation showed that these two bacteria played a crucial role in the degradation process of alkanes. However, whether they act directly on the activation of alkanes is still not clear. It is quite certain that alkanes-degrading bacteria cooperated with methanogens *Methanoculleus* formed a new methanogenic *n*-alkanes-degrading microbial community in this long-term incubation consortium. Our knowledge about the key degraders in petroleum hydrocarbon system is still quite limited under anaerobic conditions, further researches should focus on unravel of the specific role of potential alkanes-degrading bacteria.

## Author Contributions

L-YW, J-DG, and B-ZM conceived the study. BL and L-YW performed all the experiments and drafted the manuscript. ZZ helped in the experimental part and data analysis. SM was involved in the discussion on the interpretation of the results. LZ helped the analysis of GC-MS data about residual *n*-alkanes and intermediate metabolites. J-FL and S-ZY were committed to all the experiments. All authors approved the final manuscript.

## Conflict of Interest Statement

The authors declare that the research was conducted in the absence of any commercial or financial relationships that could be construed as a potential conflict of interest.

## Footnotes

1 http://web.expasy.org/translate/
