# Supplementary material for: High Frequency of Thermodesulfovibrio spp. and Anaerolineaceae in Association with Methanoculleus spp. in a Long-Term Incubation of n-Alkanes-Degrading Methanogenic Enrichment Culture
Source: Front Microbiol. 2016 Sep 16;7:1431. doi: 10.3389/fmicb.2016.01431 (PMC5025540; doi:10.3389/fmicb.2016.01431)
Supplement: Supplementary file 1 [file Presentation_1.PDF]

Supplementary Material:

High frequency of *Thermodesulfovibrio* spp. and *Anaerolineaceae* in association with *Methanoculleus* spp. in a long-term incubation of *n*-alkanes-degrading methanogenic enrichment culture

Bo Liang[1], Li-Ying Wang[1], Zhichao Zhou[2], Serge Maurice Mbadinga[1, 3], Lei Zhou[1], Jin-Feng Liu[1, 3], Shi-Zhong Yang[1, 3], Ji-Dong Gu[2], Bo-Zhong Mu[1, 3,*]

[1]State Key Laboratory of Bioreactor Engineering and Institute of Applied Chemistry, East China University of Science and Technology, Shanghai, P.R. China

[2]School of Biological Sciences, The University of Hong Kong, Pokfulam Road, Hong Kong, P.R. China

[3]Shanghai Collaborative Innovation Center for Biomanufacturing Technology, Shanghai 200237, P.R. China

*Correspondence:
Bo-Zhong Mu
State Key Laboratory of Bioreactor Engineering and Institute of Applied Chemistry, East China University of Science and Technology
Meilong Road 130, Shanghai, P.R. China
E-mail: bzmu@ecust.edu.cn
Phone: +86 21 64252063; Fax: +86 21 64252485

SUPPLEMENTARY FIGURES

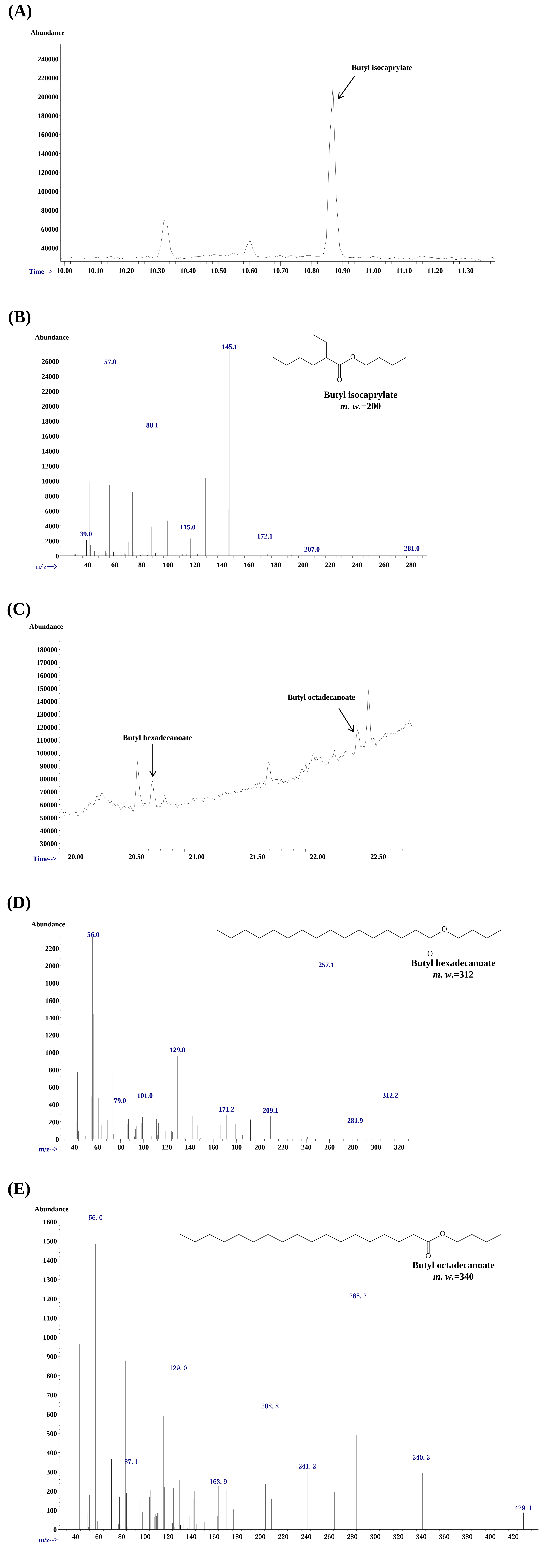

Supplementary Figure S1. The GC-MS data of long-chain fatty acids (LCFAs) that analyzed after derivatization via *n*-butyl esterification in active alkanes-degrading methanogenic enrichment cultures (T2-AE) contains gas chromatography of butyl isocaprylate (A), butyl hexadecanoate and butyl octadecanoate (C) and the relative mass spectrum of butyl isocaprylate (B), butyl hexadecanoate (D) and butyl octadecanoate (D).

Supplementary Material:

SUPPLEMENTARY FIGURES

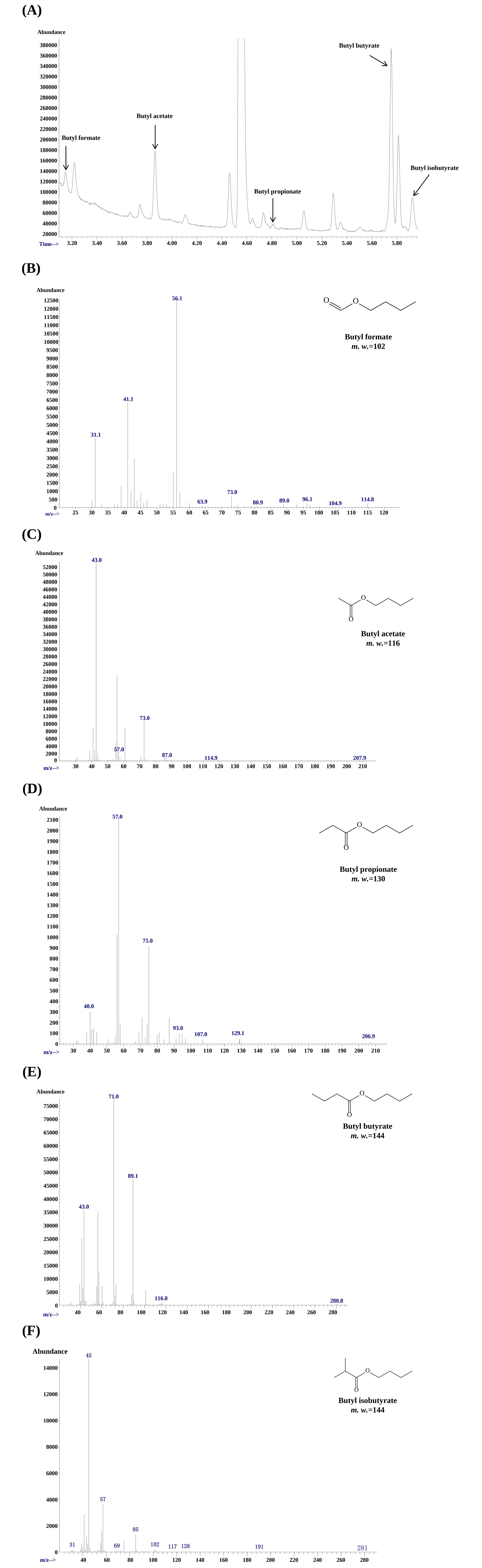

Supplementary Figure S2. The GC-MS data of volatile fatty acids (VFAs) that analyzed after derivatization via *n*-butyl esterification in active alkanes-degrading methanogenic enrichment cultures (T2-AE) contains gas chromatography of 5 volatile fatty acids (A) and the mass spectrum of butyl formate (B), butyl acetate (C), butyl propionate (D), butyl butyrate (E) and butyl isobutyrate (F).

Supplementary Material:

SUPPLEMENTARY TABLE

Supplementary Table S1. Stoichiometry of methane production in the *n*-alkanes-degrading methanogenic enrichment culture after 750 days of incubation.

Substrate	Substrate remained in the autoclaved controls (µmol)	Substrate remained in the active enrichment cultures (µmol)	Substrate consumed in the active enrichment cultures (µmol)	Theoretical Methane production (µmol)	Measured Methane production (µmol)	Percent of theoretical production
C_{15}	8.35	0.03	8.32	95.68		
C_{16}	8.55	0.58	7.98	99.75		
C_{17}	8.83	0.06	8.77	114.01		
C_{18}	9.80	0.08	9.72	133.65		
C_{19}	9.44	0.20	9.24	133.98		
C_{20}	9.44	0.15	9.29	141.67		
Sum				**718.74**	**44.76**	**6.23 %**

Note: (a) $C_{15}H_{32} + 7H_2O \rightarrow 3.5CO_2 + 11.5CH_4$; (b) $C_{16}H_{34} + 7.5H_2O \rightarrow 3.75CO_2 + 12.5CH_4$; (c) $C_{17}H_{36} + 8H_2O \rightarrow 4CO_2 + 13CH_4$; (d) $C_{18}H_{38} + 8.5H_2O \rightarrow 4.25CO_2 + 13.75CH_4$; (e) $C_{19}H_{40} + 9H_2O \rightarrow 4.5CO_2 + 14.5CH_4$; (f) $C_{20}H_{42} + 9.5H_2O \rightarrow 4.75CO_2 + 15.25CH_4$
